# Efficient derivation of embryonic stem cells from NOD-*scid Il2rg*^*−*/*−*^ mice

**DOI:** 10.1007/s13238-015-0209-6

**Published:** 2015-09-24

**Authors:** Kang Liu, Riguo Fang, Haibo Li, Weifeng Yang, Zhenchuan Miao, Jinhua Wen, Hongkui Deng

**Affiliations:** Peking University Stem Cell Research Center, Department of Cell Biology, School of Basic Medical Sciences, Peking University Health Science Center, Beijing, 100191 China; The MOE Key Laboratory of Cell Proliferation and Differentiation, College of Life Sciences, Peking-Tsinghua Center for Life Sciences, Peking University, Beijing, 100871 China; Shenzhen Stem Cell Engineering Laboratory, Key Laboratory of Chemical Genomics, Peking University Shenzhen Graduate School, Shenzhen, 518055 China; VITALSTAR Bitechnology Co., Ltd, Beijing, China

**Dear Editor,**

For decades, the stem cell field has pursued the development of humanized mice to further enhance our understanding of human hematopoiesis, innate immunity, infectious diseases, cancer biology and regenerative medicine. One significant breakthrough in generating humanized mice was the development of NOD-*scid Il2rg*^***−*/*−***^ mice, which are double homozygous for the severe combined immunodeficiency (SCID) mutation and interleukin-2Rg (*Il-2Rg*) allelic mutation (gamma c null) (Ito et al., 2002). The immunodeficient non-obese diabetic (NOD) genetic background contributes to deficiency in innate immunity with impaired dendritic cell function and macrophage activity (Shultz et al., 1995), and the *Prkdc scid* mutation leads to elimination of adaptive immunity by loss-of-function of the *Prkdc* gene, which encodes a protein critical for V(D)J recombination in T and B lymphocyte maturation (Blunt et al., 1995). Moreover, the *Il2rg*^***−*/*−***^ targeted mutation completely deletes the interleukin 2 receptor gamma chain (Il2rg), a common component of many cytokines involved in immune function such as IL-2, IL-4, IL-7, IL-9, IL-15 and IL-21, making these mice extremely deficient in NK cells which enables xenotransplantation of human cells (Cao et al., 1995). Combined, these immunodeficiency advantages make NOD-*scid Il2rg*^***−*/*−***^ mice excellent recipients to carry out *in vivo* studies of human cells, tissues and organs, especially for generating humanized mouse models, which can provide insights into *in vivo* human biology and be utilized in many pre-clinical research fields.

Although the NOD-*scid Il2rg*^***−*/*−***^ mice are highly immunodeficient and humanization on these mice has proved to be a powerful and valuable model to *in vivo* study of human disease, there are still some obvious defects in their application for generating humanized mice. For example, NOD-*scid Il2rg*^***−*/*−***^ mice lack appropriate MHC molecules for T-cell selection in the thymus and some human-specific cytokines required for engrafted human cell development and survival. Moreover, these mice also show low and variable levels of T-cell-dependent antibody responses (Shultz et al., 2007). These disadvantages might impede further application of this humanized mouse model. However, one group recently reported the acquisition of functional human embryonic stem cells-derived thymic organoids in NOD-*scid Il2rg*^***−*/*−***^ mice, providing valuable clues about the maturation process of engrafted human T cells (Sun et al., 2013). In addition, another alternative way to develop more adaptive humanized mouse models is to introduce genes, which encode human specific HLA and cytokines critical for engrafted human T cell selection and survival, to the genome of NOD-*scid Il2rg*^***−*/*−***^ mice (Shultz et al., 2007). However, this strategy still requires further genomic editing that heavily relies on the establishment of pluripotent stem cells from this mouse strain. To this end, here we report efficiently establishing embryonic stem cell lines possessing *in vitro* and *in vivo* pluripotency from NOD-*scid Il2rg*^***−*/*−***^ mice.

To derive NOD-*scid Il2rg*^***−*/*−***^ embryonic stem cell lines, a total of 28 blastocysts were recovered from NOD-*scid Il2rg*^***−*/*−***^ mice (NPG^TM^, VITALSTAR) and cultured on MEF (mouse embryonic fibroblasts) feeder cells under N2/B27 medium supplemented with the 2i/LIF, which was previously reported for use in harvesting and culturing rodent pluripotent stem cells (Ying et al., 2008). After 5 to 8 days, these 28 blastocysts were successfully cultured and showed outgrowths (Fig. 1B). These outgrowths were manually picked up and single-cell passaged by accutase, and replated onto MEF feeder cells. Since vitamin C has proved to play positive roles in promoting reprograming and maintaining pluripotency (Esteban and Pei, 2012; Shi et al., 2010), it was added into the 2i/LIF culture medium for long-term cultivation. After several passages, we successfully established 14 ES cell lines from the 28 blastocysts (Fig. 1B).

**Figure 1:**
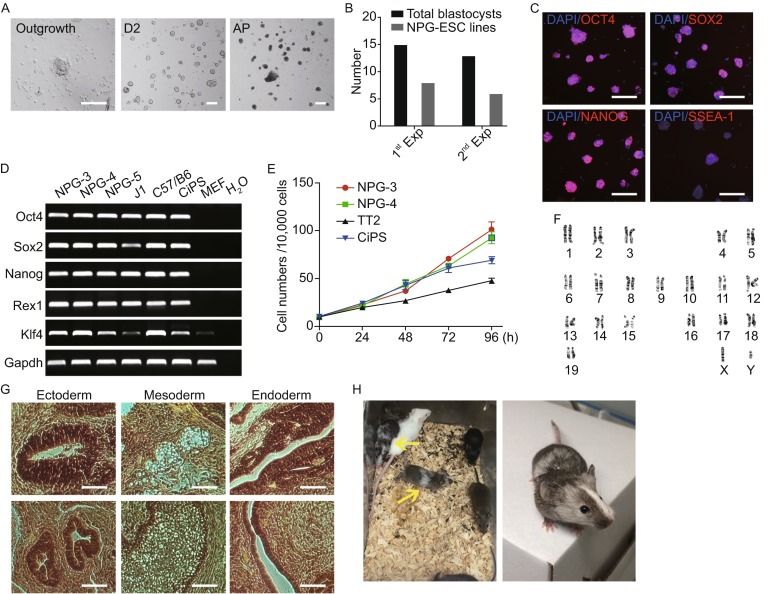
**Derivation of embryonic stem cells from NOD-** ***scid Il2rg*** ^***−*/*−***^ **(NPG) mice**. (A) Representative images of outgrowth expanded from NOD-*scid Il2rg* ^*−*/*−*^ blastocysts (left), day-2 ESC colonies after single-cell passaging (middle), day-2 ESC colonies after AP staining. Scale bar, 100 μm (B) Summary of the efficiency of NPG-ESC lines derivation from 2 separate experiments. Black columns: total blastocysts derived; Grey columns: NPG-ESC lines derived. (C) Representative images of NPG-ESC colonies immunostained for Oct4, Sox2, Nanog and SSEA-1. Scale bars, 100 μm. (D) RT-PCR analysis of pluripotency gene expression in NPG-ESCs. (NPG-3, NPG-4, NPG-5: three independently established NPG-ESC lines; J1 and C57/B6: mouse ESC lines; CiPS: chemical induced pluripotent stem cell line; MEF: mouse embryonic fibroblasts) (E) Growth curve of 2 NPG-ESC lines, 1 ESC line and 1 CiPSC line. Cells were harvested and counted at indicated time points. Starting cell number: 1 × 10^5^; 3 well replicates (F) Karyotype analysis showing the normal karyotypes of NPG-ESCs at passage 15 (G) *In vivo* teratoma differentiation of NPG-ESCs. The cells gave rise to well-differentiated mature teratomas with cells from all the three germ lineages, ectoderm, mesoderm and endoderm. Scale bars, 100 μm. (H) Chimeric mice derived from injecting C57/B6 8 cell embryos with NPG-ESCs. Yellow arrowheads indicate 2 chimeric offspring

All 14 ES cell lines showed dome-shaped colonies with smooth boundaries (Fig. 1A) and we chose 3 ES cell lines (referred to as NPG-ESCs) for further analysis. These NPG-ESCs had normal karyotypes (40, XY for male) and maintained alkaline phosphatase (AP) activity for more than 15 passages (Fig. 1A and 1F). They also grew with a doubling time similar to those of TT2 mouse embryonic stem cells (mESCs) and chemically induced pluripotent stem cells (CiPSCs) that have been established previously (Hou et al., 2013) (Fig. 1E). Moreover, the pluripotent characteristics of the NPG-ESCs were further examined by immunostaining and reverse-transcription (RT)-PCR. Specifically, NPG-ESCs were positively stained for pluripotency-specific mESCs and iPSCs markers, including Oct4, Sox2, Nanog and SSEA-1 (Fig. 1C). RT-PCR analysis showed that the NPG-ESCs also expressed typical pluripotency marker genes, such as *Oct4*, *Sox2*, *Nanog*, *Rex1* and *Klf4* (Fig. 1D). Further genomic PCR of 2 NPG-specific mutant gene sites *Il2rg* and *Prkdc* showed that both the 2 NPG ESC lines possessed NPG specific mutations at these sites (Fig. S1). Thus, these results indicate that the NPG-ESCs possess self-renewal and pluripotent characteristics *in vitro*.

To further evaluate the differentiation potential of the NPG-ESC lines, we tested their capacity to differentiate into cell types of the three germ layers. All three NPG-ESC lines chosen formed teratomas comprised of tissues from all three germ layers *in vivo* 4–5 weeks after injection into recipient mice (Fig. 1G). In addition, these NPG-ESCs were efficiently incorporated into the inner cell masses of mouse blastocysts after aggregation with eight-cell embryos. Chimeric embryos further developed into adult mice with high degrees of chimerism (Fig. 1H). Thus, these results indicate that the NPG-ESCs can fully develop and differentiate *in vivo* to form three germ layers and generate chimeric mice with germ-line contribution.

In summary, our findings provide an effective system to derive embryonic stem cell lines from NOD-s*cid Il2rg*^***−*/*−***^ mice. Moreover, unlike the traditional 2i/Lif condition which could only support NPG ESCs at early passages (Table S1), our new culture condition allows long-term maintenance of the self-renewal circuitry of NOD-*scid Il2rg*^***−*/*−***^ ESCs with normal karyotypes and *in vitro* and *in vivo* pluripotency. Efficiently establishing stable NOD-*scid Il2rg*^***−*/*−***^ ESC lines will also offer a constant and easy-to-access platform to develop new, superior humanized mouse models by further modifying the genes encoding HLA and cytokines through CRISPR/CAS9 technology in NOD-*scid Il2rg*^***−*/*−***^ mice, which will be powerful tools in pre-clinical testing and *in vivo* studies of human diseases and biological processes (Cong and Zhang 2015).

## Electronic supplementary material

Supplementary material 1 (PDF 164 kb)
