# Supplementary material for: Efficient derivation of embryonic stem cells from NOD-scid Il2rg−/− mice
Source: Protein Cell. 2015 Sep 24;6(12):916–8. doi: 10.1007/s13238-015-0209-6 (PMC4656214; doi:10.1007/s13238-015-0209-6)
Supplement: Supplementary file 1 — Supplementary material 1 (PDF 164 kb) [file 13238_2015_209_MOESM1_ESM.pdf]

Supplementary figures：

Supplementary figure 1

Geno-typing characterization of NOD-*scid Il2rg*$^{-/-}$ ESCs. Genomic PCR analysis of mutant genes specific present in NPG ESCs. Two NPG specific mutant gene sites *Il2rg* and *Prkdc* were examined by genomic PCR (C57/B6: WT ESC line; CiPS: WT chemical induced iPSC line; NPG-3, NPG-4: 2 NPG-ESC lines)

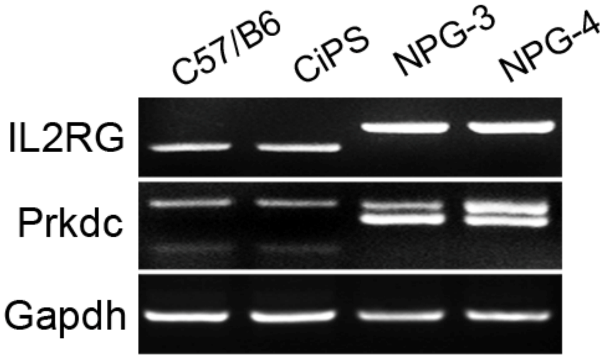

Primers for geno-typing characterization are listed below:

**Primers for geno-typing characterization**	
PRKDC-F	GAGAAAAGGAGGATCATGGATTCAAGAAATAAATGTAACG
PRKDC-WR	TGGCCCCTGCTAACTTTCTCTTAGCA
PRKDC-MF	TGGTATCCACAACATAAAATACGCTAA
PRKDC-R	CCTAAGAGTCACTTTCTCCATTTACACAGTGAAGTGCC
IL2RG-Co	GTGGGTAGCCAGCTCTTCAG
IL2RG-WT	CCTGGAGCTGGACAACAAAT
IL2RG-Mu	GCCAGAGGCCACTTGTGTAG

Supplementary tables：

Supplementary table 1

Summary of ESC lines established from NPG mice blastocysts

	2i/Lif	2i/Lif + Vc
**Blastocysts**	15	28
**Outgrowths**	12	25
**P1-P5**	9	21
**P6-P9**	2	15
**P10-P20**	0	14

Numbers indicated the amount of cell lines at each stage.

P1: passage 1

Supplementary methods：

Animals

The NOD-*scid Il2rg*$^{-/-}$(NPG^{TM}), C57BL/6 and ICR mice are housed in SPF animal facility by VITALSTAR Bitechnology Co., Ltd. All institutional and national guidelines for the care and use of laboratory animals were followed.

Derivation of ESCs form NOD-*scid Il2rg*$^{-/-}$ mice

ES cells were derived as previously describe (Evans and Kaufman, 1981; Martin, 1981). In brief, E3.5d blastocysts of NOD-*scid Il2rg*$^{-/-}$ mice were collected and seeded on MEF feeders in the basal medium K-DMEM with N2 (100x) /B27 (50x) supplements (Invitrogen), in the presence of 10ng/ml LIF (Millipore), 3uM CHIR99021 (GSK3i, Stemgent), 1uM PD0325901 (MEKi, Stemgent) and 50ug/ml Vitamin C (Sigma). The medium was changed daily until day 5 to day 8 when the outgrowth expanded. Then the outgrowths were picked up and digested by accutase (Millipore) and replated on newly prepared MEF feeder cells under the same conditions mentioned above. Notably，50ug/ml Vitamin C (Sigma) was added for stable maintenance. At around day 4 to day 5, colonies with typical mouse ESCs morphology appeared and could be manually picked for further characterization.

Cell culture

NOD-*scid Il2rg*$^{-/-}$ ES cells were cultured in the basal medium K-DMEM with N2 (100x) /B27 (50x) supplements (Invitrogen), in the presence of 10ng/ml LIF (Millipore), 3uM CHIR99021 (GSK3i, Stemgent), 1uM PD0325901 (MEKi, Stemgent) and 50ug/ml Vitamin C (Sigma). NOD-*scid Il2rg*$^{-/-}$ ES cells were single cell passaged every 4 days using accutase (Millipore) on feeder cells. The medium was changed daily.

Alkaline phosphatase (ALP) detection and immunofluorescence

To detect ALP activity, we washed the cells with phosphate-buffered saline three times and stained with BCIP/NBT (Promega) for 15 min. For immunofluorescence, the primary antibodies included those against SSEA-1 (1:50, Millipore), Nanog (1:100, R&D Systems), Oct4 (1:200, Abcam) and Sox2 (1:200, Santa Cruz Biotechnology). The secondary antibodies were rhodamine-labeled donkey anti-mouse IgG (1:100, Santa Cruz), rhodamine-labeled donkey anti-rabbit IgG (1:100, Santa Cruz Biotechnology), rhodamine-labeled goat anti-mouse IgM (1:100, Santa Cruz Biotechnology) and rhodamine-labeled donkey anti-goat IgG (1:100, Santa Cruz Biotechnology). DAPI (Roche Applied Science) was used for nuclear staining.

RT-PCR

Total RNA was isolated from cells using TRIzol (Invitrogen) and reverse

transcribed using EasyScript Reverse Transcriptase (TraNPGen Biotech) according to the manufacturer’s protocol. PCR amplification of different genes was performed using 2× EasyTaq SuperMix (TraNPGen Biotech). The primers used are listed below:

**Primers for RT - PCR**	
Oct4-S	ATGAAAGCCCTGCAGAAGGAGCTAGAAC
Oct4-A	TCTCTAGCCCAAGCTGATTGGCGATGTG
Sox2-S	TAGAGCTAGACTCCGGGCGATGA
Sox2-A	TTGCCTTAAACAAGACCACGAAA
Nanog-S	CAGGAGTTTGAGGGTAGCTC
Nanog-A	CGGTTCATCATGGTACAGTC
Klf4-S	GCGAACTCACACAGGCGAGAAACC
Klf4--A	TCGCTTCCTCTTCCTCCGACACA
Rex1-S	ACGAGTGGCAGTTTCTTCTTGGGA
Rex1-A	TATGACTCACTTCCAGGGGGCACT
Gapdh-S	CCCACTAACATCAAATGGGG
Gapdh-A	CCTTCCACAATGCCAAAGTT

Karyotype analysis

G-band chromosomal analysis was performed at the Peking University Center of Medical Genetics.

Teratoma Formation

NOD-*scid Il2rg*$^{-/-}$ ES cells were harvested and resuspended in DF12 medium with 2% Matrigel (BD Biosciences). Cells from a confluent 60-mm dish were

subcutaneously injected into a non-obese diabetes/severe-combined immunodeficient (NOD/SCID) mouse (VITALSTAR). Teratomas formed after 4-5 weeks. The teratomas were then embedded in paraffin and processed for hematoxylin and eosin staining.

Mouse embryo micromanipulation and chimera generation

For chimera generation, NOD-*scid Il2rg*$^{-/-}$ ES cells were single-cell digested by accutase (Millipore) and microinjected into 8-cell stage embryos or E3.5 blastocysts of C57 X ICR mouse embryo (10-12 cells per embryo). Approximate 15 injected embryos were transferred to each uterine horn of 2.5 days post coitum pseudo-pregnant females.

Supplementary references：

Evans, M.J., and Kaufman, M.H. (1981). Establishment in culture of pluripotential cells from mouse embryos. Nature *292*, 154-156.

Martin, G.R. (1981). Isolation of a pluripotent cell line from early mouse embryos cultured in medium conditioned by teratocarcinoma stem cells. Proc Natl Acad Sci U S A *78*, 7634-7638.
